# Linear stability analysis of chemical mechanism, Listanalchem: A tool for the search of spontaneous mirror symmetry breaking

**DOI:** 10.1016/j.mex.2023.102307

**Published:** 2023-08-16

**Authors:** Elkin Cruz, Andrés Montoya, Jesús Ágreda

**Affiliations:** a Departamento de Química, Universidad Nacional de Colombia, sede Bogotá, Colombia; b Departamento de Matemáticas, Universidad Nacional de Colombia, sede Bogotá, Colombia

**Keywords:** Homochirality, Linear stability analysis, Stoichiometric network analysis, Frank model, Frank inequality, Linear stability analysis of chemical mechanisms to find spontaneous mirror symmetry breaking

## Abstract

Homochirality, the phenomenon by which one of two virtually identical (non-superimposable mirror images) compounds is favored over the other in the chemistry of life, has been regarded as a requisite for the emergence of all living things on earth. Spontaneous mirror symmetry breaking has been proposed to produce the imbalance. Under this framework, Frank presented, in his foundational article “On spontaneous asymmetric synthesis”, a simple chemical reaction network that displays spontaneous symmetry breaking for a specific set of reaction rates. Research has since focused on finding more complex and plausible models, each one with its advantages and disadvantages. Nevertheless, finding reaction rate values that make a model exhibit spontaneous symmetry breaking is a complex task, even for specially crafted models. **LI**near **ST**ability **ANAL**ysis of **CHE**mical **M**echanism, Listanalchem, is a method and software for the search for appropriate reaction rates. It includes four different algorithms inspired by the analysis of Frank's network. Two classical algorithms are also included in Listanalchem: the Trace-Determinant plane and the Stoichiometric Network Analysis by Bruce Clarke. Listanalchem reads a chemical reaction network from plain text and runs one or more of the available algorithms according to the user selection. Listanalchem is tested and verified by studying classical, modified, and recent models proposed to explain the origin of biological homochirality.

• Listanalchem allows a fast and reliable search for instability behavior in chemical mechanisms that pretend to explain spontaneous mirror symmetry breaking.
• Listanalchem contains several model examples, including the most cited in the related literature.
• Listanalchem is a tool that tests models that pretend to explain the origin of biological homochirality, helps find errors, and aids in designing new models.

## Graphical abstract

**Figure:**
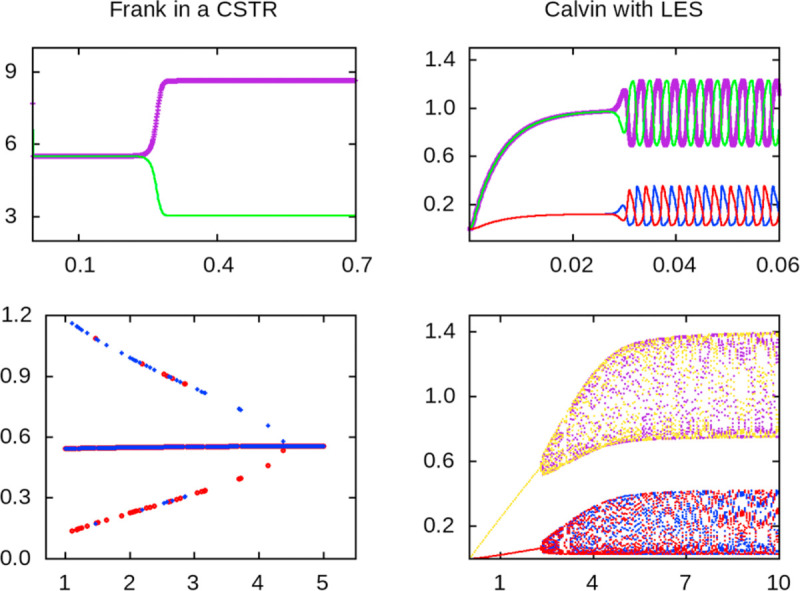

Specifications table

**Table:** 

Subject area:	Chemistry
More specific subject area:	Chemical kinetics; Chemical reaction mechanisms; Homochirality
Name of your method:	Linear stability analysis of chemical mechanisms to find spontaneous mirror symmetry breaking
Name and reference of original method:	Clarke, B. L. Stability of Complex Reaction Networks in Advances in Chemical Physics, edited by Prigogine, I., and Rice, S. A. Hoboken, NJ, USA, John Wiley & Sons, Inc., 1-215 (1980).
Resource availability:	The method as a Python computer program is available at https://gitlab.com/homochirality/listanalchem.

## Method details

 

## Introduction

Since the publication of Frank's seminal work on spontaneous symmetry breaking [1], other, more plausible, less contrived, or more realizable chemical mechanisms have been proposed as models for spontaneous symmetry breaking; for example, see references [2], [3], [4], [5]. These models, represented by a set of chemical reactions, can produce diverse behaviors besides Spontaneous Mirror Symmetry Breaking (SMSB). What makes a chemical mechanism spontaneously break the mirror symmetry, or produce any other behavior, is the particular set of reactions and their specific rate constants values, which authors often include in their work. These specific rate constants lie in a vast space of rate values, from close to zero to approximately $10^{10}$ for reactions in the liquid phase [6], and it is hard to find them by casual inspection. Furthermore, some chemical reactions that occur in several chemical mechanisms, and produce SMSB, do not necessarily follow thermodynamic or kinetic common laws [7], [8], [9], [10]. Detecting the correct rate constants, which produce SMSB and are feasible, is challenging given the complexity of the analysis involved and the usual intractability of the associated systems of differential equations. A mechanized tool that finds specific values under which the rate constants produce models with SMSB is a significant aid for testing, improving, or searching for new models.

Here, a computer program is presented to analyze models intended to explain the origin of biological homochirality and test whether the input model produces SMSB. Six algorithms are used in the analysis, starting with the most straightforward algorithm (the older and most common in literature) and finishing with the most elaborated, specific to work with complex and large reaction mechanisms with more than one enantiomeric pair. This approach makes Listanalchem versatile, efficient, and valuable to test many models in a short time, as demonstrated here throughout the analyses of 12 different models. The latter study produces significant results for each model, correcting some mistakes in some of them and, in general, serving as a guide in constructing models that are consistent thermodynamic and kinetically and capable of resembling the emergence of biological homochirality.

## Method description

Listanalchem can be downloaded freely from https://gitlab.com/homochirality/listanalchem. Detailed information on how to run the code is on that webpage and in the README.md file. Listanalchem works with both Python 2 and 3 [11]. Listanalchem uses two scientific Python libraries: NumPy [12] and SymPy [13]. Listanalchem interfaces with Reduce CAS (Reduce Computer Algebra System) [14] and its extension *redlog* [15]. Redlog is used to solve linear sets of inequalities-equalities. For example, given the set of equations x+y≤10, x≥0 and y≥0, redlog calculates that x and y must fall in the intervals: x∈[0,10] and y∈[0,10−x]. Sampling from intervals is straightforward compared to sampling from linear sets of inequalities-equalities.

Python and its libraries are easy to install in multiple Operating Systems but Reduce does not. Installing Reduce may be the most challenging component to get working Listanalchem. Please, refer to the README.md file presented with Listanalchem for details on installing all prerequisites and Listanalchem itself.

### Running Listanalchem

Running Listanalchem is simple once everything is installed. Download the code, and in its root directory, run (in the console):

python -m listanalchem –model models/Model-Name.py > Output-file-name.out

The Model-Name.py is a file composed of at least three mandatory parts: the model name, an ordered list of species names (without spaces) where the first two must be an enantiomeric pair, and a list of reactions between the species. Optionally, but recommended, the file can include the explicit pairs of reactions that must have the same rate constants, what we have called *dual pairs*. The software can detect the dual pair reactions based on the first enantiomeric pair (the first two species in the list), but if there are more than one enantiomeric pair, the extra dual pairs reactions must be introduced explicitly. Additionally, to make the command line easier to run Listanalchem, the input file could have the different options available in each of the six algorithms. All the models studied in this work have an associated input file with the recommended structure, added with comments, and they are on the models folder that comes with Listanalchem. Those files can be used to verify what is presented here and as templates for analyzing other (maybe new) models not tested here.

The command line presented above saves the output of Listanalchem in the file *Output-file-name.out*. The user can make a folder to save the outputs of Listanalchem and give that path in the command line; for example, if the user creates a new folder called outputs, inside the folder where Listanalchem resides, the command line will be:

*python -m listanalchem –model models/Model-Name.py > outputs/Output-file-name.out*

It is important to mention that if the model under study has more than one pair of enantiomers, that fact will not be critical for the first five algorithms. Those extra pairs of enantiomers will not be considered (as extra pairs of enantiomers) for the analysis. In the sixth algorithm, specially designed for models with more than one enantiomeric pair, the enantiomeric pairs must be introduced as an ordered list. That extra information simplifies the stability matrix (the matrix to be analyzed). In this respect, one requirement of the list of dual pairs in case more than one couple must be equal (three, four, or more like in the output flows of a continuous stirred tank Reactor, CSTR) is that those pairs must have the same element as the first one. The second element will be one of the others that are equal, i.e. (1,2), (1,3), (1,4), ... , and so on.

On the other hand, no matter what the analysis is, Listanalchem always calculates the stoichiometric matrix, the reaction order matrix, the velocity function vector (a function per reaction), and the system of differential equations according to the mass action law. These data are key to all analyses and passed on to other functions as the basis for calculations.

Currently, Listanalchem is stable and functional and can help study any model related to SMSB.

### Implemented algorithms

Listanalchem has six algorithms, which are included in the folder listanalchem/analyses after the program's installation. These six algorithms are:

1. **Trace-Determinant plane.** The first algorithm computes the symbolic *Jacobian matrix* together with its *trace* and *determinant* from the *differential equations* that define the network. The trace-determinant plane analyzes linear systems of two differential equations, and only two species can be involved in the reaction mechanism [16,17], which limits its applicability and generality.
2. **Stoichiometric Network Analysis (SNA).** SNA is well-defined in the literature [18], [19], [20]. SNA reduces the Jacobian matrix to a smaller matrix called the current matrix with linear entries, and it then applies a heuristic to determine under which circumstances (reaction rate values) there is SMSB. Our version of SNA has an additional sampling step to find values that satisfy the restrictions created by the SNA algorithm. As a result, this analysis returns specific concentrations and reaction rate constants for a given chemical reaction network, such that when simulated, the SMSB is evident [21].
3. **Six-categories.** The third algorithm focused on the 2×2 Jacobian of a chiral network as defined in reference [22]. The reactions of the analyzed mechanism are classified into six categories, each with a specific role in the symmetry-breaking process. This algorithm requires that all reactions of the mechanism under study fall under one of the six categories; if it does not, the analysis is impossible and will not be executed.
4. **Frank inequality – Nonlinear.** The fourth algorithm is based on a condition occurring in the *Frank network* that creates symmetry-breaking states. This condition is nicknamed the *Frank inequality* [23]. All that is needed to find homochiral states in a chemical mechanism that has them is to sample solutions of the *Frank inequality* related to the network and explicitly computed by the algorithm. Unfortunately, because *Frank inequality* comes from nonlinear elements in the Jacobian Matrix, the inequality becomes a nonlinear one that is not easy to sample, i.e., there is a simple formula to find homochiral states, but finding values that fulfill this simple nonlinear formula is hard. We want to remark that, until we find a robust way to sample nonlinear inequalities, we are limited to admiring the simplicity of the inequality and wonder what to do.
5. **Frank Inequality plus SNA**. This algorithm combines the *Frank inequality* with the current matrix obtained from SNA. This fifth algorithm works as a mixture of the second and fourth algorithms. Although this analysis is more involved than the previously presented, it has given us far better results on almost all networks we have analyzed, including lower computation times.
6. **Frank Inequality plus SNA plus networks with more than one enantiomeric pair**. An extension of the previous algorithm to networks with two or more enantiomeric pairs. This algorithm uses the previously mentioned tools, but the matrix to be analyzed is constructed by the difference between the two submatrices representing the whole set of left and right enantiomers presented in the model [24].

## In-depth description of the method with examples

Listanalchem offers six algorithms to analyze whether homochirality can arise from a chemical reaction mechanism. To study a mechanism, all Listanalchem requires is to define that mechanism in an input plain text file as those available in the folder models created after Listanalchem installation, like the example below, which represents the Kondepudi-Nelson model [2].

modelname = ‘Kondepudi-Nelson’

species = [‘L’, ‘D’]

reactions = [

"      <-> L  ", # 0 & 1.

"      <-> D  ", # 2 & 3.

"L     <-> 2 L", # 4 & 5.

"D     <-> 2 D", # 6 & 7.

"L + D  ->    ", # 8.

]

analyses = {

"trace-determinant"   : { ######### First algorithm.  #########

          "enabled"               : True,

           "2by2-jacobian"         : True,

          "num-samples"           : 10,

          "plot"                  : True,

"time-to-show-plot"     : 0.3

       },

"sna"                 : { ######### Second algorithm.  #########

           "enabled"              : True,

           "dual-pairs-in-ec"      : False,

          ``instability-heuristic'' : "mineurs", # or: "trace-determinant", "characteristic-polynomial",

           "sum-mineurs"           : True,

           "max-mineur-search-stop": 5,

           "simplification-tries"  : 10000,

           "num-samples"           : 10,

           "samples-folder"        : ‘samples/Kondepudi-Nelson-SNA’,

"six-categories"    : { ######### Third algorithm.  #########

           "enabled"               : True,

           "num-samples"           : 10,

"samples-folder"        : ‘samples/Kondepudi-Nelson-3A-6c’

       },

"frank-ineq-nonlinear": { ######### Fourth algorithm.  #########

"enabled"               : True

       },

"frank-ineq-linear"   : { ######### Fifth algorithm.  #########

           "enabled"               : True,

           "dual-pairs-in-ec"      : True,

           "num-samples"           : 10,

           "samples-folder"        : ‘samples/Kondepudi-Nelson-5A’,

"samples-for-proportion": 10000

       },

"frank-pseudoquiral"  : { ######### Six algorithm.  #########

           "enabled"               : True,

           "enantiomeric-pairs"    : [(0, 1)],

           "dual-pairs-in-ec"      : False,

           "instability-heuristic" : "mineurs", # or: "trace-determinant", "characteristic-polynomial",

           "sum-mineurs"           : True,

           "max-mineur-search-stop": 5,

           "simplification-tries"  : 10000,

           "num-samples"           : 10,

           "samples-folder"        : ‘samples/Kondepudi-Nelson-6A’,

}

}

Listanalchem parses the plain text and generates the stoichiometric and reaction order matrices [18], [19]–20,25,26], which are, together, an alternative form to define a reaction mechanism. From these two matrices, it is easy to obtain the differential equations that characterize the temporal evolution of the reaction network.

As an example of what Listanalchem needs as entry and what it computes, let us analyze the Kondepudi-Nelson (KN) model [2,27,28], defined in the input plain text file presented above^1^.

The model in the input file, like the one presented above, is analyzed after running the following code in a command console:

                       *python -m listanalchem –model models/Kondepudi-Nelson.py*

The basic computations made by Listanalchem are:

**Table:** 

Species: [’L’, ’D’]	Reactions list: [ ’ -> L’, ’ -> D’, ’L -> ’, ’D -> ’, ’L -> 2 L’, ’D -> 2 D’, ’2 L -> L’, ’2 D -> D’, ’L + D -> ’]	Dual pair equations (equations are numbered from 0 to 8): [(0, 1), (2, 3), (4, 5), (6, 7)] Regular reactions (not dual) [8],

$$\text{Stoichiometric}\;\text{Matrix}:\;S=\left[\begin{array}{ccccccccc} 1 & 0 & -1 & 0 & 1 & 0 & -1 & 0 & -1\\ 0 & 1 & 0 & -1 & 0 & 1 & 0 & -1 & -1 \end{array}\right]$$

$$\text{Reactions}\;\text{Order}\;\text{Matrix}:\;K=\left[\begin{array}{ccccccccc} 0 & 0 & 1 & 0 & 1 & 0 & 2 & 0 & 1\\ 0 & 0 & 0 & 1 & 0 & 1 & 0 & 2 & 1 \end{array}\right]$$

Velocity Function^2^: $v^{⊤}=\left[\begin{array}{ccccccccc} k_{0} & k_{1} & k_{2}x_{0} & k_{3}x_{1} & k_{4}x_{0} & k_{5}x_{1} & k_{6}x_{0}^{2} & k_{7}x_{1}^{2} & k_{8}x_{0}x_{1} \end{array}\right]$

Differential equations functions (polynomials) vector: $\dot{x}=\left[\begin{array}{c} k_{0}-k_{2}x_{0}+k_{4}x_{0}-k_{6}x_{0}^{2}-k_{8}x_{0}x_{1}\\ k_{1}-k_{3}x_{1}+k_{5}x_{1}-k_{7}x_{1}^{2}-k_{8}x_{0}x_{1} \end{array}\right]$

The latter can be obtained in latex format, adding to the previous command line the argument *–latex t*

*python -m listanalchem –latex t –model models/Kondepudi-Nelson.py*

Notice that from here onward, the species (L, D) will be named $\left(x_{0},\;\;x_{1}\right)$. We rename them because this is how they are treated in the implementation.

The following subsections present an overview of each algorithm. For additional explanations, please check the corresponding references.

### First algorithm: trace-determinant plane

The Trace-Determinant plane is a method to determine the instability of a linear dynamic system with only two variables [16,17]. The stability (instability) is determined based on the trace and the determinant of the 2×2 matrix that describes the system. A linear system is unstable and has saddle points if its determinant is negative and real-valued. When the determinant is positive, the system will be unstable if the trace is positive. In any other case, the system is stable except when the determinant, the trace, or the discriminant are zero. The particular cases when determinant, trace, or discriminant are equal to zero are bifurcation points. Those points have a very low probability of being sampled using pseudo-random number generation, as our algorithm does because they constitute a small region compared to the whole bidimensional plane.

Linear Stability Analysis formulates that a nonlinear dynamic system can be analyzed as a linear one if we stay close to the steady states. In a steady state, the concentrations of the species remain constant as functions of time, and it means that all the associated differential equations get equal to zero. Linear Stability Analysis also indicates that, in a steady state, one can analyze the system dynamics using the Jacobian of the corresponding differential equations [16,17].

The Trace-Determinant plane algorithm of Listanalchem computes the Jacobian's trace, determinant, and discriminant of the chemical model (a mechanism) under study.

Notice that Listanalchem does not implement, in this first algorithm, a procedure to find suitable rate constants from the above conditions (differential equations equal to zero, positive variables, and Jacobian's trace, determinant, and discriminant). The latter is because there is no efficient algorithm to sample from nonlinear sets of equations [29]. Instead, arbitrary pseudo-random values are assigned to the rate constants and 0.01 (in arbitrary units) to the initial concentrations of the enantiomers (the only two species) of the model under study. Then, the result is presented as a point in the Trace-Determinant plane.

This algorithm is not new but included in Listanalchem as the starting point to analyze the whole problem. The computations made by this algorithm are helpful for beginners and to have a complete vision of the smallest models (only two variables) able to generate homochirality as the Frank or Kondepudi-Nelson models.

### Execution example

The results of running the first algorithm on the Kondepudi-Nelson network are:

• The Jacobian from the differential equations:
  $$J=\left[\begin{array}{cc} -k_{1}+k_{4}-2k_{5}x_{0}-k_{8}x_{1} & -k_{8}x_{0}\\ -k_{8}x_{1} & -k_{3}+k_{6}-2k_{7}x_{1}-k_{8}x_{0} \end{array}\right]$$
• The Jacobian trace:
  $$T_{J}=-k_{1}-k_{3}+k_{4}-2k_{5}x_{0}+k_{6}-2k_{7}x_{1}-k_{8}x_{0}-k_{8}x_{1}$$
• The Jacobian determinant:
  $$\begin{array}{c} D_{J}=k_{1}k_{3}-k_{1}k_{6}+2k_{1}k_{7}x_{1}+k_{1}k_{8}x_{0}-k_{3}k_{4}+2k_{3}k_{5}x_{0}+k_{3}k_{8}x_{1}+k_{4}k_{6}-\\ 2k_{4}k_{7}x_{1}-k_{4}k_{8}x_{0}-2k_{5}k_{6}x_{0}+4k_{5}k_{7}x_{0}x_{1}+2k_{5}k_{8}x_{0}^{2}-k_{6}k_{8}x_{1}+2k_{7}k_{8}x_{1}^{2} \end{array}$$
• The Jacobian discriminant:
  $$\begin{array}{c} dis_{J}=4k_{8}^{2}x_{0}x_{1}-4\left(k_{1}-k_{4}+2k_{5}x_{0}+k_{8}x_{1}\right)\left(k_{3}-k_{6}+2k_{7}x_{1}+k_{8}x_{0}\right)+\\ {\left(k_{1}+k_{3}-k_{4}+2k_{5}x_{0}-k_{6}+2k_{7}x_{1}+k_{8}x_{0}+k_{8}x_{1}\right)}^{2} \end{array}$$
• The Jacobian's eigenvalues and eigenvectors. They are too long to show here, but they are available as the output of Listanalchem.

Then, the algorithm assigns pseudo-random values to k’s and 0.01 to x’s, plots a Trace-Determinant plane using the latter values, and establishes the stability of the network. The output text of Listanalchem can be saved in a file by adding to the main command line code, as shown before, the symbol “>” and then the path and name of the file where the user wants to save the results; for example (if the user has created the folder *outputs*),

*python -m listanalchem –model models/Kondepudi-Nelson.py > outputs/K-N.out*

The specific model generated in this way can be tested using Chemkinlator [30] or any other software capable of making chemical kinetics simulations.

The same procedure used for the first algorithm, command line code, and options can be used for the other five algorithms. In any case, the user can explore other options, such as standard commands to execute computer programs (or specifically python) from the command console, and more advisable, to use the files that come with Listanalchem in the folder models. Those files can be used as templates for new models and to explore the options available. These options for each algorithm, as presented in the above example input file for the Kondepudi-Nelson model, can be changed intuitively; for example, from True to False, or from a particular number to another more convenient according to the user and the model under analysis.

#### Second algorithm: Stoichiometric Network Analysis (SNA)

The Stoichiometric Network Analysis (SNA) is an algorithm to analyze the stability of steady states following three stages:

1. Computation of the matrix called current matrix, V(J), which comes from the factorization of the Jacobian matrix. The V(J) matrix is less complex than the Jacobian. The entries of V(J) are linear functions, an important fact because the entries of the Jacobian matrix are nonlinear. Clarke suggested this transformation, and it is explained in detail in the literature [18,19,21].
2. The search for negative terms in the determinant of matrix V(J). There are three options to do this, Trace-determinant, only possible when the V(J) is a 2×2 matrix as in the previous algorithm; mineurs according to the references [20,25,26,31]; or characteristic-polynomial. If there are no negative terms, the reaction network is stable, and the computation ends. Otherwise, the equalities and inequalities that must be satisfied are printed, and the next step is executed.
3. A sample of the set of states that fulfill the above conditions is computed.

If the conditions are correct and the sampling process works fine, the result is a set of rate constants that produces SMSB in the analyzed model.

#### Important notes about the implementation of the SNA in Listanalchem

Some remarks must be made about the particular implementation of SNA in Listanalchem, which are presented in the following sections.

### Clarke transformation

Clarke found that the Jacobian of a reaction network, restricted to only steady states, can be described by the multiplication of four matrices:

J=S·diag(E·j)·K·[diag(h)]=V(J)·[diag(h)].

Clarke noted that the matrix [diag(h)] does not alter the result of the stability analysis; therefore, it can be ignored in the analysis. Then, the stability study of a network can be reduced to the analysis of the called current matrix V(J)=S·diag(E·j)·K, which is the matrix studied in Listanalchem.

#### Extreme currents

The computation of the extreme currents, E, is a crucial step before the calculation of the current matrix V(J). The extreme currents computation in Listanalchem was borrowed from that of COPASI [31]. Also, the algorithm of Schmitz et al. [20] was tested to confirm the results.

#### Extended stoichiometric matrix

Listanalchem can force the dual reaction restrictions into the convex system, i.e., Listanalchem can encode which reaction rates must be equal directly into V(J). For this, before the computation of the extreme currents, Listanalchem can, as an option, extends the stoichiometric matrix with rows that encode the dual pairs. The number of extreme currents got smaller in all the trials where the extended stoichiometric matrix was used to compute the extreme currents. It was also noticed that the fewer extreme currents a reaction mechanism has, the less resource intensive the analysis becomes. Therefore, every time the restrictions to the convex system are applied, an important reduction in computing time is observed.

#### Conditions for unstable states in the current matrix V(J)

Listanalchem second algorithm (SNA) implements three strategies to find conditions that encode unstable states: Trace-determinant, mineurs, and characteristic-polynomial. If the matrix V(J) is of size 2×2, we can apply the trace-determinant method, the same as in the previous algorithm. In case V(J) is bigger than 2×2, two heuristics can be used to determine the stability: mineurs, and characteristic-polynomial.

The mineurs heuristic is based on the assumption that computing the characteristic polynomial of the matrix is too expensive or too complex to work with. To find the mineurs, the heuristic proposes the computation of coefficients blocks of the characteristic polynomial that may be related to instability. Only negative coefficients in the characteristic polynomial give chances to find positive roots for the characteristic polynomial and then the possibility of instabilities. Therefore, the mineur heuristic defines the conditions for instability from a partial computation of the characteristic polynomial of the current matrix. This heuristic is helpful in the case of large current matrices but may produce false positive unstable states (states that are not unstable) and false negative (the algorithm does not find unstable states although they exist).

The characteristic-polynomial heuristic computes the characteristic polynomial of the current matrix, char, and defines the condition for instability as char<0. This heuristic warrants that, if it is fulfilled, at least one real and positive eigenvalue for the current matrix exists, which suggests instability in the network. However, the characteristic-polynomial option takes longer computation times than the mineurs option; and then, when complex and large models are analyzed, the computer system could get stuck.

### Sampling process

Even though there is no generalized and efficient algorithm to sample from a semialgebraic or a nonlinear set of equations, there are algorithms to sample linear sets of equations, like the one used by Reduce [14], the software used in Listanalchem to sample those linear sets. Then, Listanalchem implements a heuristic algorithm that replaces variables for random values to transform a set of nonlinear equations into a linear set. This heuristic does not warrant a successful sampling. Nonetheless, the heuristic could sample unstable steady states for the networks we tested. On the other hand, some networks require more resources to be analyzed, given their complexities; and we have found that networks with minimal sets of extreme currents performed best. Then, we recommend applying the option to force the conditions of the dual pairs into the computation of the extreme currents, as this procedure showed to reduce the number of computed extreme currents. The user can add this option to the input file by writing the line: *“*dual-pairs-in-ec*”: True*, as seen in the files available inside the folder models of Listanalchem or the example input file above.

The final result of the execution of this analysis is either a message by Listanalchem indicating its inability to find unstable steady states or a sample of, hopefully, unstable steady states. The number of states that will be sampled can be selected using *“num-samples”: 10*,. Those samples can be optionally saved in text files with “.json” format using the line *“samples-folder”: 'samples/File-name-to-save-samples'*. Those files also have a “.simu.” pre-extension and can be read by Chemkinlator [30] to obtain graphical time series and bifurcation diagrams.

### Execution example

The more relevant results of running the second algorithm (SNA) analysis on the Kondepudi-Nelson network (without forcing the dual pair rate restrictions on the extreme currents) are (the complete set of results, with the different options, can be seen after running Listanalchem):

• Extreme Currents Matrix**E**(computed from Stoichiometric Matrix**S**). Each column is an extreme current vector.
  $$E=\;\left[\begin{array}{cccccccccccc} 1 & 0 & 0 & 0 & 1 & 0 & 0 & 0 & 1 & 0 & 1 & 0\\ 0 & 1 & 0 & 0 & 0 & 0 & 1 & 0 & 1 & 1 & 0 & 0\\ 1 & 0 & 1 & 0 & 0 & 0 & 0 & 0 & 0 & 0 & 0 & 0\\ 0 & 1 & 0 & 1 & 0 & 0 & 0 & 0 & 0 & 0 & 0 & 0\\ 0 & 0 & 1 & 0 & 0 & 1 & 0 & 0 & 0 & 1 & 0 & 1\\ 0 & 0 & 0 & 1 & 0 & 0 & 0 & 1 & 0 & 0 & 1 & 1\\ 0 & 0 & 0 & 0 & 1 & 1 & 0 & 0 & 0 & 0 & 0 & 0\\ 0 & 0 & 0 & 0 & 0 & 0 & 1 & 1 & 0 & 0 & 0 & 0\\ 0 & 0 & 0 & 0 & 0 & 0 & 0 & 0 & 1 & 1 & 1 & 1 \end{array}\right]$$
• E_omega_prim matrix. This is a*diag*(**E∙j**), and here it is written as a vector.
  $$diag\left(E\cdot j\right)=\left[\begin{array}{c} j_{0}+j_{10}+j_{4}+j_{8}\\ j_{1}+j_{6}+j_{8}+j_{9}\\ j_{0}+j_{2}\\ j_{1}+j_{3}\\ j_{11}+j_{2}+j_{5}+j_{9}\\ j_{10}+j_{11}+j_{3}+j_{7}\\ j_{4}+j_{5}\\ j_{6}+j_{7}\\ j_{10}+j_{11}+j_{8}+j_{9} \end{array}\right]$$
• V(J) Matrix.
  $$V\left(J\right)=\left[\begin{array}{cc} -j_{0}-j_{10}-2j_{4}-j_{5}-j_{8} & -j_{10}-j_{11}-j_{8}-j_{9}\\ -j_{10}-j_{11}-j_{8}-j_{9} & -j_{1}-2j_{6}-j_{7}-j_{8}-j_{9} \end{array}\right]$$
• Restrictions to solve, which includes:
• $j_{i}>0$
• The dual pair restrictions:
  $$\begin{array}{c} j_{0}+j_{10}+j_{4}+j_{8}=j_{1}+j_{6}+j_{8}+j_{9}\\ j_{0}+j_{2}=j_{1}+j_{3}\\ j_{11}+j_{2}+j_{5}+j_{9}=j_{10}+j_{11}+j_{3}+j_{7}\\ j_{4}+j_{5}=j_{6}+j_{7} \end{array}$$
• The polynomial to solve which can come from one of three options: *“mineurs”, “characteristic-polynomial”* or *“trace-determinant”*. The latter is only possible when the V(J) is a 2×2 matrix. The desired option can be selected using the command *“instability-heuristic” : "mineurs",* where *“mineurs”* can be changed for one of the other two options. In our example, for the Kondepudi-model and using the option *“mineurs”*, the polynomial is:
  $$\begin{array}{c} -j_{0}j_{1}-2j_{0}j_{6}-j_{0}j_{7}-j_{0}j_{8}-j_{0}j_{9}-j_{1}j_{10}-2j_{1}j_{4}-j_{1}j_{5}-j_{1}j_{8}+j_{10}^{2}+2j_{10}j_{11}\\ \;\;-2j_{10}j_{6}-j_{10}j_{7}+j_{10}j_{8}+j_{10}j_{9}+j_{11}^{2}+2j_{11}j_{8}+2j_{11}j_{9}-4j_{4}j_{6}-2j_{4}j_{7}\\ \;\;-2j_{4}j_{8}-2j_{4}j_{9}-2j_{5}j_{6}-j_{5}j_{7}-j_{5}j_{8}-j_{5}j_{9}-2j_{6}j_{8}-j_{7}j_{8}+j_{8}j_{9}+j_{9}^{2}> \end{array}$$
• Finally, the algorithm samples $j_{i}$ values that fulfill the previous restrictions if the polynomial is linear. Otherwise, the heuristic method mentioned before is used to obtain linear expressions to be sampled. The user can define the number of samples to take using the option *“num-samples”: 10,* in this case, 10. The $j_{i}$ values are converted into rate constants, $k_{i}$, assigning pseudo-random concentrations to the involved species between 0.001 to 0.01 in arbitrary units, and using the relationship v=E·j, see references [18], [19], [20] for details.

As mentioned before, running this algorithm with the following option *“dual-pairs-in-ec”: True,* force the dual pair rate restrictions on the computation of the extreme currents resulting in a smaller quantity of extreme currents. We can see below the substantial reduction in the number of extreme currents when we compute them with the extended stoichiometric matrix:

• Extended Stoichiometric matrix. A new row has been added for every dual pair (four dual pairs = four new rows). Compare with the previous stoichiometric matrix, which only has two rows.
  $$S_{Extended}=\left[\begin{array}{ccccccccc} 1 & 0 & -1 & 0 & 1 & 0 & -1 & 0 & -1\\ 0 & 1 & 0 & -1 & 0 & 1 & 0 & -1 & -1\\ 1 & -1 & 0 & 0 & 0 & 0 & 0 & 0 & 0\\ 0 & 0 & 1 & -1 & 0 & 0 & 0 & 0 & 0\\ 0 & 0 & 0 & 0 & 1 & -1 & 0 & 0 & 0\\ 0 & 0 & 0 & 0 & 0 & 0 & 1 & -1 & 0 \end{array}\right]$$
• New extreme currents matrix:

$\;E_{S_{Extended}}=\left[\begin{array}{cccccc} 1 & 0 & 1 & 0 & 1 & 0\\ 1 & 0 & 1 & 0 & 1 & 0\\ 1 & 1 & 0 & 0 & 0 & 0\\ 1 & 1 & 0 & 0 & 0 & 0\\ 0 & 1 & 0 & 1 & 0 & 1\\ 0 & 1 & 0 & 1 & 0 & 1\\ 0 & 0 & 1 & 1 & 0 & 0\\ 0 & 0 & 1 & 1 & 0 & 0\\ 0 & 0 & 0 & 0 & 1 & 1 \end{array}\right]$ ; a 9 × 6 matrix, with only half columns compared to the previous.

• The E_omega_prim also changes:
  $$diag{\left(E\cdot j\right)}_{S_{Extended}}=\left[\begin{array}{c} j_{0}+j_{2}+j_{4}\\ j_{0}+j_{2}+j_{4}\\ j_{0}+j_{1}\\ j_{0}+j_{1}\\ j_{1}+j_{3}+j_{5}\\ j_{1}+j_{3}+j_{5}\\ j_{2}+j_{3}\\ j_{2}+j_{3}\\ j_{4}+j_{5} \end{array}\right]$$
• The new V(J) matrix has, of course, smaller elements compared to the previous:
  $$V{\left(J\right)}_{S_{Extended}}=\left[\begin{array}{cc} -j_{0}-2j_{2}-j_{3}-j_{4} & -j_{4}-j_{5}\\ -j_{4}-j_{5} & -j_{0}-2j_{2}-j_{3}-j_{4} \end{array}\right]$$

Then, the computation follows as was shown in the preceding example.

Notice the symmetry in the new current matrix $V{\left(J\right)}_{S_{Extended}}$. This symmetry also exists in the original matrix but is hidden by the non-explicit equivalences of the dual pair conditions. This symmetry will play an essential role in the criterion called *Frank inequality* [23], which is crucial in developing the last three algorithms.

### Third algorithm: six-categories

This algorithm performs all the steps defined in the reference [22]. The general idea of this algorithm is to classify each reaction of the mechanism under study into one of six categories, which are crucial for a mechanism capable of producing SMSB. Each category has a role in the occurrence of symmetry-breaking states. This algorithm can analyze any network constructed by combining reactions from the six categories; otherwise, it cannot. The six categories are presented below, in a generic form on the left, together with our example test model (Kondepudi-Nelson) on the right:

**Table:** 

$R_{1}$: Synthesis:	Kondepudi-Nelson model
$\nu_{i1}^{\left(-\right)}X_{1}+\cdots +\nu_{in}^{\left(-\right)}X_{n}\to L+\nu_{i1}^{\left(+\right)}X_{1}+\cdots +\nu_{in}^{\left(+\right)}X_{n}$	$A+B\;\overset{k_{0}}{\to }\;L$
$\nu_{i1}^{\left(-\right)}X_{1}+\cdots +\nu_{in}^{\left(-\right)}X_{n}\to D+\nu_{i1}^{\left(+\right)}X_{1}+\cdots +\nu_{in}^{\left(+\right)}X_{n}$	$A+B\;\overset{k_{1}}{\to }\;D$
$R_{2}$: fo-Decomposition:	
$L+\nu_{i1}^{\left(-\right)}X_{1}+\cdots +\nu_{in}^{\left(-\right)}X_{n}\to \nu_{i1}^{\left(+\right)}X_{1}+\cdots +\nu_{in}^{\left(+\right)}X_{n}$	$L\;\overset{k_{2}}{\to }\;A+B$
$D+\nu_{i1}^{\left(-\right)}X_{1}+\cdots +\nu_{in}^{\left(-\right)}X_{n}\to \nu_{i1}^{\left(+\right)}X_{1}+\cdots +\nu_{in}^{\left(+\right)}X_{n}$	$D\;\overset{k_{3}}{\to }\;A+B$
$R_{3}$: Autocatalytic:	
$L+\nu_{i1}^{\left(-\right)}X_{1}+\cdots +\nu_{in}^{\left(-\right)}X_{n}\to 2L+\nu_{i1}^{\left(+\right)}X_{1}+\cdots +\nu_{in}^{\left(+\right)}X_{n}$	$A+B+L\;\overset{k_{4}}{\to }\;2L$
$D+\nu_{i1}^{\left(-\right)}X_{1}+\cdots +\nu_{in}^{\left(-\right)}X_{n}\to 2D+\nu_{i1}^{\left(+\right)}X_{1}+\cdots +\nu_{in}^{\left(+\right)}X_{n}$	$A+B+D\;\overset{k_{5}}{\to }\;2D$
$R_{4}$: so-Decomposition:	
$2L+\nu_{i1}^{\left(-\right)}X_{1}+\cdots +\nu_{in}^{\left(-\right)}X_{n}\to L+\nu_{i1}^{\left(+\right)}X_{1}+\cdots +\nu_{in}^{\left(+\right)}X_{n}$	$2L\;\overset{k_{6}}{\to }\;A+B+L$
$2D+\nu_{i1}^{\left(-\right)}X_{1}+\cdots +\nu_{in}^{\left(-\right)}X_{n}\to D+\nu_{i1}^{\left(+\right)}X_{1}+\cdots +\nu_{in}^{\left(+\right)}X_{n}$	$2D\;\overset{k_{7}}{\to }\;A+B+D$
$R_{5}$: no-Enantioselective:	
$L+\nu_{i1}^{\left(-\right)}X_{1}+\cdots +\nu_{in}^{\left(-\right)}X_{n}\to L+D+\nu_{i1}^{\left(+\right)}X_{1}+\cdots +\nu_{in}^{\left(+\right)}X_{n}$	∅
$D+\nu_{i1}^{\left(-\right)}X_{1}+\cdots +\nu_{in}^{\left(-\right)}X_{n}\to L+D+\nu_{i1}^{\left(+\right)}X_{1}+\cdots +\nu_{in}^{\left(+\right)}X_{n}$	
$R_{6}$: Inhibition:	
$L+D+\nu_{i1}^{\left(-\right)}X_{1}+\cdots +\nu_{in}^{\left(-\right)}X_{n}\to \nu_{i1}^{\left(+\right)}L+\nu_{i2}^{\left(+\right)}D+\nu_{i1}^{\left(+\right)}X_{1}+\cdots +\nu_{in}^{\left(+\right)}X_{n}$	$L+D\;\overset{k_{8}}{\to }\;P$

The symbols L and D represent enantiomers, $X_{i}$, A and B represent arbitrary mixtures of chemical species, and $\nu_{i}^{\left(-\right)},\nu_{i}^{\left(+\right)}$ are the stoichiometric coefficients of reaction, negative ${}^{\left(-\right)}$ for reagents and positive ${}^{\left(+\right)}$ for products, which are small, possibly zero, integers.

Montoya et al. [22] have shown that for homochirality to occur, this kind of reaction mechanism (six categories) must contain autocatalytic and inhibition reactions. Notice the existence of $R_{3}$: Autocatalytic and $R_{6}$: Inhibition reactions in the Kondepudi-Nelson network, which guarantees symmetry-breaking states.

The third algorithm determines whether the network can contain symmetry-breaking states and, if it has, computes a sample of those states, producing files with a set of rate constants that solve the set of equalities and inequalities proposed in reference [22]. Those files, as before, have *.json format and can be read by Chemkinlator [30] to make the respective simulations (time series and bifurcation diagrams). Notice that the *.json are open standard format files that the users can take for chemical kinetic simulations with their preferred software, probably making the required modifications (if necessary) to the *.json results files.

It is important to mention that the analysis of the Frank model using this algorithm allowed us to find a necessary condition for the appearance of symmetry-breaking states. We have called this condition the *Frank Inequality*, which comes from a particular characteristic of the Jacobian matrices of the reaction mechanisms capable of producing spontaneous mirror symmetry breaking [22]. The *Frank Inequality* will be used in the subsequent algorithms.

### Execution example

One of the most relevant features of the third algorithm is related to the symmetry of the Jacobian matrix. Consider the Jacobian matrix J of the Kondepudi-Nelson network, where all the dual pair reaction rates are equaled:

$$J=\left[\begin{array}{cc} -k_{2}+k_{4}-2k_{6}x_{0}-k_{8}x_{0} & -k_{8}x_{0}\\ -k_{8}x_{0} & -k_{2}+k_{4}-2k_{6}x_{0}-k_{8}x_{0} \end{array}\right]$$

Notice that $J_{11}=J_{22}$ and $J_{12}=J_{21}$. This symmetry can be seen in all chemical mechanisms capable of SMSB. From this remark and using some results from linear stability analysis, it is possible to demonstrate that a symmetry-breaking state should hold [22]:

$$\begin{array}{ccc} J_{11}-J_{12}>0 & \Rightarrow & -k_{2}+k_{4}-2k_{6}>0\\ J_{11}+J_{12}<0 & \Rightarrow & -k_{2}+k_{4}-2k_{6}-2k_{8}<0 \end{array}$$

The inequality $J_{11}-J_{12}>0$ is what we call the *Frank inequality*.

On the other hand, from this third algorithm (the six categories), it should hold:

D+DE−C−E−A−P=0,

where P, D, A, DE, and E are the summation of the reaction rates for each pair of reactions from the synthesis, fo-decomposition, autocatalytic, so-decomposition, and no-enantioselective respectively; and, C is the summation of the reaction rates of all the inhibition reactions multiplied by the corresponding stoichiometric coefficients [22]. In our example:

$$-k_{0}+k_{2}-k_{4}+k_{6}+k_{8}=0$$

With these linear restrictions in place, the third algorithm can use a Computer Algebra System, in our case Reduce [14], as the previous algorithm had done, to find a set of values for the reaction rates that define a set of breaking symmetry states.

The final result of the analysis gives us the following values for the Kondepudi-Nelson model:

$$\overset{k_{0}}{\to }L\;\;\;\;\;\overset{k_{1}}{\to }D\;\;\;k_{0}=\;k_{1}=k_{2}-k_{4}+k_{6}+k_{8}$$

$$L\overset{k_{2}}{\to }\;\;\;\;D\overset{k_{3}}{\to }\;\;\;k_{2}=k_{3}=\left(0,\infty \right)$$

$$L\;\overset{k_{4}}{\to }2L\;\;\;D\;\overset{k_{5}}{\to }2D\;\;k_{4}=\;k_{5}=\left(k_{2},\infty \right)$$

$$2L\overset{k_{6}}{\to }L\;\;\;2D\;\overset{k_{7}}{\to }D\;\;k_{6}=\;k_{7}=\left(0,\;-\frac{k_{2}}{2}+\frac{k_{4}}{2}\right)$$

$$\;\;\;L+D\overset{k_{8}}{\to }\;\;\;k_{8}=\;\left(max\left(-k_{2}+k_{4}-k_{6},\;-\frac{k_{2}}{2}+\frac{k_{4}}{2}-k_{6}\right),\;\infty \right)$$

What is left to do is to sample a single point from the ranges above and test if it is indeed unstable, using the generated files with the *.simu.json format and Chemkinlator [30] (or any other similar software), as mentioned before.

## Fourth algorithm: Frank inequality - Nonlinear

*Frank Inequality* is essential in searching symmetry-breaking states: it encodes a necessary condition for homochirality [22]. Even though *Frank Inequality* is not enough to ensure homochirality, many states that fulfill this condition also show homochiral behavior.

This fourth algorithm outputs the *Frank Inequality* that a steady state must fulfill to be unstable and capable of producing SMSB, together with a list of equations and inequalities that describe the model under study.

### Execution example

Following the Kondepudi-Nelson example, this analysis outputs:

• Differential equations equal to zero:
  $$k_{0}-k_{1}x_{0}+k_{4}x_{0}-k_{5}x_{0}^{2}-k_{8}x_{0}^{2}=0$$
• The determinant of the Jacobian should be different from zero:
  $$-k_{8}^{2}x_{0}^{2}+\left(-k_{1}+k_{4}-2k_{5}x_{0}-k_{8}x_{0}\right)\left(-k_{3}+k_{6}-2k_{7}x_{0}-k_{8}x_{0}\right)\neq 0$$
• Frank inequality.

$\;-k_{1}+k_{4}-2k_{5}x_{0}>0$

Unfortunately, all equations are nonlinear; thus, we cannot easily sample their solutions.

## Fifth algorithm: Frank inequality + SNA

*Frank Inequality* provides us with a necessary condition, a criterion, for symmetry-breaking states. However, as discussed in the previous paragraph, this criterion is insufficient for sampling symmetry-breaking states, given the complexity of sampling arbitrary nonlinear sets.

To overcome the nonlinearity problem, we use the transformation proposed by Clarke, which is used in the SNA (second) algorithm. Remember that Clarke's transformation factorizes the Jacobian matrix into four factors. Three of them, grouped in the current matrix V(J), are enough to study the stability of the chemical mechanism. The current matrix V(J) has linear entries instead of the usual nonlinear entries of the Jacobian matrix, and it is also restricted to steady states; thus, the conditions that must be checked in the previous fourth algorithm get reduced to only one condition: *Frank Inequality*; and because *Frank Inequality* is constructed from the current matrix, V(J), the inequality is linear and can be easily sampled.

### Execution example

Recall the current matrix V(J) for the Kondepudi-Nelson network shown in the second algorithm - SNA example:

$$V\left(J\right)=\left[\begin{array}{cc} -j_{0}-2j_{2}-j_{3}-j_{4} & -j_{4}-j_{5}\\ -j_{4}-j_{5} & -j_{0}-2j_{2}-j_{3}-j_{4} \end{array}\right]\;.$$

The fifth algorithm computes from the current matrix V(J) the *Frank Inequality*:

$$V{\left(J\right)}_{11}-V{\left(J\right)}_{12}=-j_{0}-2j_{2}-j_{3}+j_{5}>0,$$

and finds suitable values for the js using the Computer Algebra System Reduce [14]:

$$j_{0}=\left(0,\infty \right);j_{1}=\left(0,\infty \right);\;j_{2}=\left(0,\infty \right);j_{3}=\left(0,\infty \right);j_{4}=\left(0,\infty \right);j_{5}=\left(\text{max}\left(0,j_{0}+2j_{2}+j_{3}\right),\infty \right).$$

Listanalchem generates, from this solution, random samples of $j_{i}$ values, which are used to obtain the respective rate constants, $k_{i}$, assigning concentrations to the involved species between 0.001 to 0.01 (arbitrary units) and considering the relationship v=E·j, as was made before when the second algorithm was described.

## Sixth algorithm: Frank inequality + SNA + networks with more than one enantiomeric pair

In any chemical mechanism that contains a single pair of enantiomers capable of producing SMSB, one can observe that the 2×2 submatrix of the Jacobian that encodes the behavior of the two enantiomers is a symmetric matrix. We get *Frank Inequality* from the analysis of those submatrices. Montoya et al. [24] showed a similar pattern in the Jacobian matrices of chemical mechanisms with more than one enantiomeric pair. The pattern encompasses not four cells in the matrix but four subregions of the matrix (submatrices), and this algorithm operates over these subregions. Analogous to chemical mechanisms with only one enantiomeric pair, it can take out both submatrices from the Jacobian and subtract one from the other.

The result of applying the *Frank Inequality* to the current matrix V(J) from a chemical mechanism with more than one enantiomeric pair is not a single expression (inequality); the result is a matrix of size m×m, where m is the number of enantiomeric pairs in the network. This new matrix, $V^{\prime }\left(J\right)$, encodes all steady states that may break the symmetry. From here onward, we can use the same machinery of the SNA analysis previously used to find a sample of those symmetry-breaking states. Notice that, even though we are still using SNA to sample unstable states, the matrix to analyze is considerably smaller (m<n), where n is the number of total species in the mechanism including enantiomeric and non-enantiomeric species; then, the size of the matrix to be analyzed is reduced by a factor no greater than ½.

Also, it is important to notice that the preceding five algorithms do not explicitly consider the extra pairs of enantiomers. The previous fact makes possible the analysis of those chemical mechanisms with more than one pair of enantiomers that sometimes failed to be analyzed with pure SNA, generally due to the enormous size of the mechanism.

Notice that this new matrix, $V^{\prime }\left(J\right)$ can be analyzed with all the standard techniques presented above to find instabilities, and remember that one of those analyses is SNA, the one chosen here, and it works well.

### Execution example

The Kondepudi-Nelson network has only one enantiomeric pair. Thus, we use in this section a different model, the Replicator of Hochberg and Ribó [32], defined as:

$$\begin{array}{ccc} A+{}^{1}R_{D}+{}^{2}R_{D}\overset{k_{0}}{\underset{k_{1}}{⇋}}2^{1}R_{D}+{}^{2}R_{D} & & A+{}^{1}R_{L}+{}^{2}R_{L}\overset{k_{2}}{\underset{k_{3}}{⇋}}2^{1}R_{L}{+}^{2}R_{L}\\ A{+}^{2}R_{D}{+}^{1}R_{D}\overset{k_{4}}{\underset{k_{5}}{⇋}}2^{2}R_{D}{+}^{1}R_{D} & & A{+}^{2}R_{L}{+}^{1}R_{L}\overset{k_{6}}{\underset{k_{7}}{⇋}}2^{2}R_{L}{+}^{1}R_{L}\\ {}^{1}R_{D}\overset{k_{8}}{\to }\emptyset & & {}^{1}R_{L}\overset{k_{10}}{\to }\emptyset \\ {}^{2}R_{D}\;\overset{k_{9}}{\to }\emptyset & & {}^{2}R_{L}\overset{k_{11}}{\to }\emptyset \\ & \bar{\emptyset }\;\overset{k_{12}}{\to }\;A\\ & A\;\overset{k_{13}}{\to }\;\emptyset \end{array}$$

The most relevant facts related to this model, and the most relevant results about it that are obtained after running the sixth algorithm, are listed below:

• Chemical species (enantiomers and non enantiomers): ${}^{1}R_{D}{,}^{1}R_{L}{,}^{2}R_{D}{,}^{2}R_{L}$, *A*.
• Dual pair reactions: {(0,2),(1,3),(4,6),(5,7),(8,9),(8,10),(8,11),(8,13)}
• Regular reactions (not dual): {12}
• V(J) matrix reshaped to show clearly the symmetry [AB;BA] in matrix:
  $$\left[\begin{array}{ccccc} -j_{0} & j_{1} & 0 & 0 & j_{0}+j_{1}\\ j_{1} & -j_{0} & 0 & 0 & j_{0}+j_{1}\\ 0 & 0 & -j_{0} & j_{1} & j_{0}+j_{1}\\ 0 & 0 & j_{1} & -j_{0} & j_{0}+j_{1}\\ j_{0}-2j_{1} & j_{0}-2j_{1} & j_{0}-2j_{1} & j_{0}-2j_{1} & -4j_{0}-5j_{1} \end{array}\right]$$
  $$A\;\;\;\;\;-\;\;\;\;\;B\;\;\;\;\;=\;\;\;\;\;V^{{}^{\prime }}\left(J\right)$$
  $$\;\left[\begin{array}{cc} -j_{0} & j_{1}\\ j_{1} & -j_{0} \end{array}\right]\;\;-\;\;\left[\begin{array}{cc} 0 & 0\\ 0 & 0 \end{array}\right]\;=\;\left[\begin{array}{cc} -j_{0} & j_{1}\\ j_{1} & -j_{0} \end{array}\right]$$

Notice the considerable dimension reduction between the analysis of the original current matrix V(J) with a characteristic polynomial of degree five versus the new current matrix $V^{\prime }\left(J\right)$ with a characteristic polynomial of degree two.

One of the main advantages of this dimension reduction, in this case to a 2×2 matrix, is that we can use the well-known Trace-Determinant plane analysis, the first algorithm of Listanalchem. Then, using the option *“instability-heuristic” : “trace-determinant”,* the sixth algorithm tells us:

• Trace: $-2j_{0}$, determinant: $j_{0}^{2}-j_{1}^{2}$, discriminant: $4j_{1}^{2}$.
• Trace-determinant says that the instability is possible when: determinant<0 or determinant>0 and trace>0.
• Restrictions to solve $\left[j_{0}>0,j_{1}>0,-j_{0}^{2}+j_{1}^{2}>0\right]$.

The latter equations can be sampled using the same heuristic mentioned in the SNA analysis. A solution output example is:

• (Randomly) Chosen variable values: $j_{0}=1.17511584853$.
• The values that the remaining variables can take, given the already chosen (see above) values for variables $j_{0}$: $j_{1}=\left(1.17512,\infty \right)$ .

From these results, a sample of the rate constants $k_{i}$ is computed as it was made before.

## Some additional considerations

Some notes must be added to clarify the results that can be obtained with Listanalchem. The notes are classified into two sets. The first is about the six algorithms implemented, and the second is about the most relevant results obtained after analyzing classical and recent models proposed to explain the origin of biological homochirality. The second set also includes Trapp et al.'s mechanism that explains the Soai reaction's behavior [33].

### The algorithms

#### The first algorithm: the Trace-Determinant plane

The Trace-Determinant plane was added to Listanalchem to have a complete set of tools. It is restricted to models with only two species, but it is useful when a large and complex model can be reduced to a 2×2 matrix, which is possible when the second, fifth, and sixth algorithms are used, thanks in part to the extended stoichiometric matrix.

#### The second algorithm: stoichiometric network analysis (SNA)

The second algorithm, SNA, also has a previous history, but we have improved it by introducing the extended stoichiometric matrix, which allows us to simplify the expressions for calculations and sampling. Remarkably, when we add to the stoichiometric matrix rows that code the duality relation between reactions, we get a substantial size reduction in the number of extreme currents matrix and then in the size of the matrix that is tested for stability, the so-called current matrix. This means that some models, too complex or large to be analyzed by SNA without the extended stoichiometric matrix, can now be studied. For example, the Iwamoto model [3], the imperfect version, has an extreme current matrix of size 20×47, while the same matrix computed using the extended stoichiometric matrix is only 20×4. This is a significant reduction that makes the calculations possible because the analysis gets stuck without that. Another important and particular addition to the traditional SNA algorithm is the function *nonlinear_to_linear* (inside the Listanalchem file sampler.py) which solves the problem of sampling nonlinear inequalities [29]. This heuristic procedure finds the most frequent variable involved in the nonlinear inequalities and replaces it with a pseudo-random number. The process is repeated until a linear inequality is reached. This latter linear inequality is solved using Reduce (redlog) [14,15].

#### The third algorithm: the six categories

The third algorithm relies on a syntactical analysis of the requirements a model must fulfill to produce SMSB: such a model must have at least one autocatalytic reaction and at least one inhibition reaction [22]. This is the core of many models proposed to explain the biological homochirality origin, which is the case of the Frank model [1]. However, this third algorithm is restricted to models that are entirely constituted by reactions within a restricted set of reactions. Only six types of reactions constitute this latter set, and the algorithm is not general. On the other hand, in some models, inhibition reactions have been replaced by the called Limited EnantioSelective reactions (LES) [34], which play the role of the inhibition reactions and produce negative feedback [35,36]. LES reactions are one of the six categories included in the aforementioned scheme. LES reactions are included neither in the Frank nor the Kondepudi-Nelson model, which were an important source for this algorithm. Nevertheless, adding LES reactions is not enough to get a general algorithm.

#### The fourth algorithm: the Frank inequality - nonlinear

The fourth algorithm uses nonlinear algebra to extend the basic ideas of the previous algorithm. The result is a semialgebraic constraint called *Frank inequality* [22,23]. Unfortunately, it cannot sample sets of rate constants from those equations due to the nonlinearities of the involved semialgebraic constraints. Then, this algorithm is presented here as a starting point for future work.

#### The fifth algorithm: the Frank inequality + SNA

The fifth algorithm partially solves the *nonlinearity problem* of the previous algorithm, searching for instabilities not in the Jacobian matrix but in the current matrix, whose entries are all linear. Then, *Frank inequality* is applied to the current matrix, and the sampling is done over the convex space spanned by the currents and not in the space of rate constants. Then, a suitable sample of rate constants is possible by using v=E·j and concentrations between 0.001 and 0.01 in arbitrary units. This algorithm combines SNA (second algorithm) and *Frank inequality*.

#### The sixth algorithm, the Frank inequality + SNA for models with more than one enantiomeric pairs

Although the previous algorithms can analyze many models intended to explain the origin of biological homochirality, they have problems with models with more than one enantiomeric pair. In those models, which usually have many species and reactions, the first five algorithms cannot obtain a result, and usually get stuck due to the large size of the matrices involved. The sixth algorithm extends the *Frank inequality* to those models with two or more enantiomeric species. Montoya et al. [24] have noticed that the symmetry found in the Jacobian matrix of models with only one enantiomeric pair, for example, the one presented for the Kondepudi-Nelson model, is also found in models with two or more enantiomeric pairs, but now, the symmetry is not between elements of the matrix but between submatrices that represents the whole subset of *left* and *right* species, as presented before in the replicator model of Hochberg and Ribó [32].

### The models

The user of Listanalchem can test several models or variations of one single model. Listanalchem offers six algorithms to test the capability of one model to generate spontaneous mirror symmetry breaking. The algorithms can handle different models, but they are not always successful in doing their job. However, if one algorithm cannot analyze a particular model, one of the others can probably do the job. The user of Listanalchem must find the best algorithm for each specific model. In the following, we present the most relevant results from analyzing some of the most representative models proposed to explain the origin of biological homochirality.

#### The Frank model

The Frank model has serious flaws related to the basic principles of thermodynamics and kinetics because details are not explicit. An explicit Frank model consistent with the basic principles of thermodynamics and kinetics is presented below [21].

$$\begin{array}{ccc} & & \;\overset{\;\;\;k_{f_{0}}\;}{\to }A\;\;\;\;\overset{\;\;\;k_{f_{1}}\;}{\to }B\\ & & A\;+\;B\;\;\overset{k_{2}}{\underset{k_{3}}{⇋}}\;\;L\;\;\;A\;+\;B\;\;\overset{k_{4}}{\underset{k_{5}}{⇋}}\;D\\ & & A\;+\;L\;\;\;\overset{k_{6}}{\underset{k_{7}}{⇋}}\;\;2L\;\;\;A\;+\;D\;\;\overset{k_{8}}{\underset{k_{9}}{⇋}}\;\;\;2D\\ & & \;L+D\;\;\overset{k_{10}}{\underset{k_{11}}{⇋}}\;\;P\;\\ & & \;\;\;A\;\overset{\;\;\;k_{f_{12}}\;}{\to },B\;\overset{\;\;\;k_{f_{12}}\;}{\to },L\;\overset{\;\;\;k_{f_{12}}\;}{\to },D\overset{\;\;\;k_{f_{12}}\;}{\to },P\;\overset{\;\;\;k_{f_{12}}\;}{\to }\;.\; \end{array}$$

The latter model is available as the file “Frank-Rev-P-AB-CSTR.py” on the *models* folder of Listanalchem. This model includes only reversible reactions, which is not always the case with the models that occur in the literature. The model is an open system because of the explicit continuous well stirred tank reactor (CSTR) [37] represented by the pseudo-reactions of input, for example $\underset{\to }{k_{f_{0}}}$*A*, and output, for example *A* $\underset{\to }{k_{f_{12}}}$. The addition of those reactions makes the system of differential equations gets larger, with a larger Jacobian matrix, and then the model becomes harder to analyze. The use of Listanalchem has allowed us to handle this model, and more than that, it allowed us to test several previous versions of the model until we arrived at this final version. All this work was possible thanks to the aid of Listanalchem. Observe also that this model is an improved version of the Kondepudi-Nelson model and can be used to make explicit the requirements for a chemical model to be able to produce SMSB: autocatalysis (positive feedback), inhibition, or limited enantioselectivity reactions (negative feedback) and the inflow and outflow of reagents.

#### Models with limited enantioselectivity (LES) reactions

The models of Iwamoto [3], APED [4], and limited enantioselectivity (LES) [34] contain a particular type of reactions that are called *imperfect* by Iwamoto and which accounts for some “defects”, “errors” or “limits” of the autocatalytic reactions. These reactions do not generate two units of the same autocatalytic enantiomer but one unit of each enantiomer, as can be seen in the following reactions of the LES model:

$\mathrm{Auto}\mathrm{cata}\mathrm{lysis}:\;\;\;A\;+\;L\;\;\underset{k_{-i}}{\overset{k_{i}}{⇋}}\;\;\;L\;+\;L\;;\;\;\;\;\mathrm{Limi}\mathrm{ted}\;\mathrm{Enan}\mathrm{tioS}\mathrm{elec}\mathrm{tivi}\mathrm{ty}\;\left(\mathrm{LES}\right):\;\;\;A\;+\;L\;\;\underset{k_{-j}}{\overset{k_{j}}{⇋}}\;\;\;L\;+\;\;D\;\;.$ .

In the mentioned three models, SMSB is possible if the rate constants of the autocatalytic reactions are different from those of the LES reactions, $k_{i}\neq k_{j}$ and $k_{-i}\neq k_{-j}$. This can be tested using Listanalchem, the input file Blanco-et-al-LES-2013.py, and setting the respective pair of reactions constants in the input file equal (SMSB is not possible):

dual_pairs=[(0,2),(1,3),(4,6),(5,7),(4,8),(4,10),(5,9),(5,11)]; or not equal (SMSB is possible):

dual_pairs=[(0,2),(1,3),(4,6),(5,7),(8,10),(9,11)].

This fact holds true in the Iwamoto model, perfect and imperfect versions, and the APED model. This latter model does not contain an explicit autocatalytic reaction, but it contains three different sets of dimerization reactions that are intended to produce delayed autocatalysis. Those three sets of reactions are constituted by four reactions, grouped into two pairs. Both reactions in a pair are assigned the same rate constant, and the complementary pair is assigned the same rate constant but multiplied by a parameter that controls the difference between those two constants; for example,

$$L^{*}\;+\;L\;\overset{p}{\to }\;LL\;,\;\;D^{*}\;+\;L\;\overset{\alpha p}{\to }\;DL\;,\;\;L^{*}\;+\;D\;\overset{\alpha p}{\to }\;LD\;,\;\;D^{*}\;+\;D\;\overset{p}{\to }\;DD.$$

It can be checked that α cannot be equal to 1 if SMSB is desired. It is not clear why those pairs of rate constants must be different, as it is not clear why the analogous inequalities satisfied by the Iwamoto and LES models must be satisfied. This exciting fact will not be discussed here because it is not the main subject of this work, but it must be remarked that Listanalchem was very useful in discovering and testing this fact.

The Iwamoto, APED, and LES models are available as input files to Listanalchem in the folder *models*.

#### The Calvin model added with LES reactions

The Calvin model, proposed by the Nobel Prize Melvin Calvin in 1969 [38], is stable. However, if we add to it inhibition or LES reactions, we get SMSB. The process can be seen as adding the proper reactions to the basic model. There are at least three versions of the Calvin model, and several modifications are possible for each. Here, we are interested in those modifications that include LES reactions. Below is one of those possibilities, corresponding to an open system throughout a CSTR, and is available as the input file *Calvin-2-CSTR-LES.py*.

$$\begin{array}{cc} {\left[NR_{3}\right]}_{0}\;\overset{\;\;\;k_{f_{0}}\;\;\;}{\to }\;NR_{3} & {\left[Allyl-X\right]}_{0}\;\overset{\;\;\;k_{f_{1}}\;\;\;}{\to }\;Allyl-X\\ NR_{3}+\;Allyl-X\;\overset{k_{2}}{\underset{k_{3}}{⇋}}L-A & NR_{3}+Allyl-X\;\;\overset{k_{4}}{\underset{k_{5}}{⇋}}\;\;D-A\\ L-A\;\;\overset{k_{6}}{\underset{k_{7}}{⇋}}L-B & D-A\;\;\overset{k_{8}}{\underset{k_{9}}{⇋}}\;\;D-B\\ L-A\;+\;L-B\;\;\;\overset{k_{10}}{\underset{k_{11}}{⇋}}\;\;2\;L-B & D-A\;+\;D-B\;\;\overset{k_{12}}{\underset{k_{13}}{⇋}}\;\;2\;D-B\\ L-A\;+\;L-B\;\;\;\overset{k_{14}}{\underset{k_{15}}{⇋}}\;\;L-B\;+\;D-B & D-A\;+\;D-B\;\;\;\overset{k_{16}}{\underset{k_{17}}{⇋}}\;\;D-B\;+\;L-B\\ L-A\;\overset{k_{f_{s}18}}{\to }\;\;,\;\;D-A\;\overset{k_{f_{s}19}}{\to }\;\;,\;\;L-B\;\overset{k_{f_{s}20}}{\to } & D-B\;\overset{k_{f_{s}21}}{\to }\;\;,\;\;NR_{3}\;\overset{k_{f_{s}22}}{\to }\;\;,\;\;Allyl-X\;\overset{k_{f_{s}22}}{\to } \end{array}$$

**Fig. 1:**
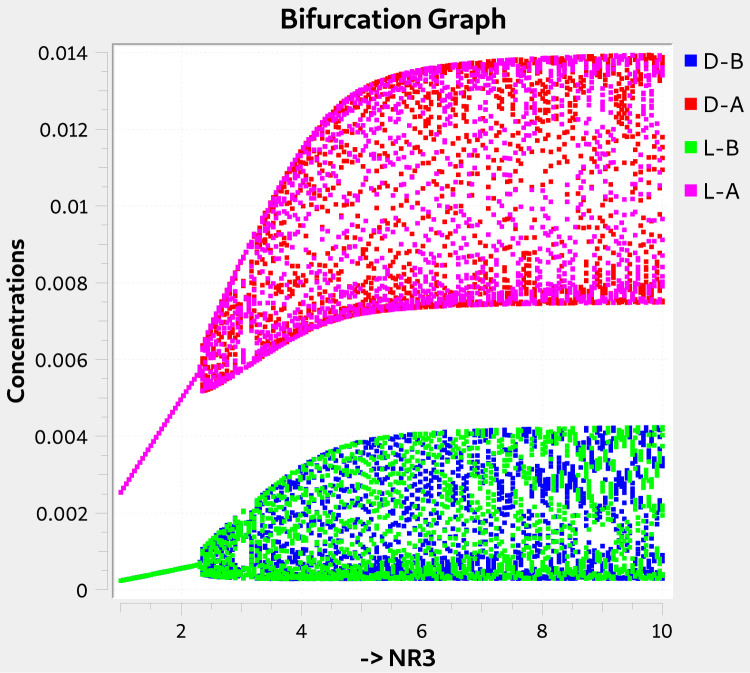
The Calvin model, version 2, in a CSTR and added with LES reactions. The figure shows the Chemkinlator bifurcation window with the bifurcation diagram. The classical chaos is evident.

Apart from SMSB, this model exhibits chaotic behavior, as represented by the typical chaotic bifurcation diagram in Fig. 1.

It is important to remark that, as with the previous three models, the rate constants of the LES reactions must differ from the rate constants of the autocatalytic reactions; otherwise, SMSB or chaos becomes impossible.

Other versions of the Calvin Model are available in the *models* folder of Listanalchem.

##### Models with instabilities that do not produce homochirality

The Frank model with all reactions reversible and explicit product P, input file Frank-Rev-P.py, is stable. However, observe that our fifth algorithm sample some unstable states but also observe the message 0 sampled points are frank (which means that they do not produce SMSB). Additionally, those unstable states require that some rate constants turn to zero, which means that those reactions are not part of the model, then it is a new model. These results can be verified by numerical simulations using Chemkinlator [30] or similar software. In this case, the unlimited growth in the concentration of the enantiomers is evident. Also, the user can review the results of the other algorithms to get clues and confirm the particular behavior of one model. In the example under study, the second algorithm tells us that the Frank-Rev-P.py model is stable, which implies that SMSB does not occur.

Similarly, the Kondepudi-Nelson model with all the species made explicit, file Kondepudi-Nelson-AS.py holds unstable states, which is true because the product grows infinitely, which means instability. Remember that linear stability searches for unstable states which can be incapable of producing SMSB. Then, this is not an error; it is just the theoretical limitation of linear stability analysis applied to this problem. However, the other algorithms that can work with this model, the second and fifth, tell us that there is no symmetry-breaking states (no SMSB).

#### Recent models

Some recent models published between 2019 and 2020 are also available in the models folder of Listanalchem: the replicator model of Hochberg and Ribó [32], the chaotic replicator of Hochberg, Torralba, and Morán [39], the revised model of Kondepudi-Nelson applied to a model activated by radiation [40], the kinetic model for asymmetric autocatalysis (Scheme 8) of Blackmond [41], and the chemical mechanism for the Soai reaction proposed by Trapp et al. [33].

Our software confirms the results of those authors, which is a way to validate Listanalchem, but more important, using Listanalchem to test these recent models helps us to find errors and improve those models. Specifically, we have found an error in the first reaction of the Kondepudi-Mundy model that must be T+Π⇄TE instead of S+Π⇄TE; this can be tested easily using Listanalchem.

Also, we have found that the kinetic model for asymmetric autocatalysis (Scheme 8) of the 2020’s review of Blackmond:

$$\begin{array}{cc} A+Z\;\;\overset{k_{0}}{\to }\;\;R & A+Z\;\;\overset{k_{0}}{\to }\;\;S\\ A+Z+R\;\;\overset{k_{\mathrm{mon}}}{\to }\;\;R+\;R & A+Z+S\;\;\overset{k_{\mathrm{mon}}}{\to }\;\;S+\;S\\ A+Z+\mathrm{RR}\;\;\overset{k_{\mathrm{homo}}}{\to }\;\;R\;+\;\mathrm{RR} & A+Z+\mathrm{SS}\;\;\overset{k_{\mathrm{homo}}}{\to }\;\;S+\;\mathrm{SS}\\ A+Z+\mathrm{SR}\;\;\overset{k_{\mathrm{hete}\mathrm{ro}}}{\to }\;\;R+\;\mathrm{SR} & A+Z+\mathrm{SR}\;\;\overset{k_{\mathrm{hete}\mathrm{ro}}}{\to }\;\;S\;+\;\mathrm{SR}\;\;, \end{array}$$

which is not capable of producing SMSB can be modified, in the following way, to guarantee SMSB

$$\begin{array}{ccc} A\;+\;Z\;\;\overset{k_{0}}{\underset{k_{1}}{⇋}}\;\;R & & A\;+\;Z\;\;\overset{k_{2}}{\underset{k_{3}}{⇋}}\;\;S\\ A+Z+R\;\;\overset{k_{4}}{\underset{k_{5}}{⇋}}\;\;R+R & & A+Z+S\;\;\overset{k_{6}}{\underset{k_{7}}{⇋}}\;\;S+S\\ R+R\;\;\overset{k_{8}}{\underset{k_{9}}{⇋}}\;\;RR & & S+S\;\;\overset{k_{10}}{\underset{k_{11}}{⇋}}\;\;SS\\ & R+S\;\;\underset{k_{13}}{\overset{k_{12}}{⇋}}\;\;\mathrm{SR}\\ A+Z+RR\;\;\overset{k_{14}}{\underset{k_{15}}{⇋}}\;\;R+RR & & A+Z+SS\;\;\overset{k_{16}}{\underset{k_{17}}{⇋}}\;\;S+SS\\ A+Z+\mathrm{SR}\;\;\underset{k_{19}}{\overset{\;k_{18\;}}{⇋}}\;\;R+\;\mathrm{SR} & & A+Z+\mathrm{SR}\;\underset{k_{21}}{\overset{\;k_{20\;}}{⇋}}\;S+\;\mathrm{SR}\;\;. \end{array}$$

The above model is available in the input file Blackmond-Rev-2020.py of the folder models of Listanalchem. Observe that scheme 8 of Blackmond, probably not intended to be a model of SMSB, contains irreversible reactions for the reagents (precursors) A and Z but not a reaction to produce the dimers RR, SS, and SR. Starting from that scheme, we constructed the model presented above, which makes all the reactions reversible and is enriched with explicit reactions that produce the dimers. Other versions of that scheme 8 can be considered, but we wanted to point out how Listanalchem helps in the construction of models that are consistent, explicit, and capable of producing SMSB. Also, it is important to mention that only the sixth algorithm could find SMSB in the proposed model.

Concerning the Trapp et al. mechanism for the Soai reaction, Listanalchem can manage it despite its huge size, 34 reactions (counting forwards and backwards), and 26 species, with seven enantiomeric pairs. The results show that the mechanism is stable, as presented in the reference [33], with some irreversible reactions. However, if all reactions are made reversible, as they must be, the fifth algorithm found sets of rate constants that produce the SMSB, although the other algorithms do not. The latter is a remarkable result considering the mentioned huge size of the model and the many options that a model of that size can have. Once again, it demonstrates how Listanalchem can help improve those proposed models.

Finally, we would like to observe that most papers are dedicated to analyzing a single model (see all the references cited). Here we have explored several models. Moreover, we were able to detect flaws and suggest improvements for some of them. This was possible with the aid of Listanalchem.

## Summary

Listanalchem implements six algorithms to analyze chemical mechanisms intended to explain the origin of biological homochirality. The algorithms search for spontaneous mirror symmetry breaking in the input models. Each successive algorithm builds upon the previous one. The first algorithm is the well-known Trace-Determinant plane, the starting point. The second algorithm is an improved version of the stoichiometric network analysis (SNA) of Bruce Clarke. It has been specially adapted to search for homochirality, and the most relevant improvement is the addition of rows to code the duality relation between reactions, which reduces the size of the extreme current matrix and then the complexity of the matrix to be analyzed. The third algorithm, six categories, takes advantage of the structure of the Frank, Kondepudi-Nelson, and LES models to create a set of six types of reactions that can be used to build a model capable of producing SMSB. The analysis of these models makes evident what we have called the *Frank Inequality*, which is used in the fourth to sixth algorithms. The fourth algorithm makes explicit the power of the *Frank Inequality* to find the instability regions of a model, resumed in a set of equalities and inequalities. Unfortunately, that set is nonlinear, and no readily available algorithm exists to solve those coupled equations. This difficulty was surpassed by combining the *Frank Inequality* and the SNA, which allows working with the convex parameters to obtain linear expressions that are possible to sample. However, in complex mechanisms, the convex parameters can be mixed nonlinearly. In that case, we have used a heuristic that works most of the time, even with large and complex models, and which linearizes the aforementioned nonlinear systems. The sixth algorithm uses the tools developed before to work with models with more than one enantiomeric pair, and it takes advantage of the symmetry of the Jacobian matrix generated when there are more than one enantiomeric pair. The latter makes it possible to obtain nontrivial results related to large models that are hard to analyze with the first five algorithms.

Listanalchem has been tested and used to analyze the more representative models that pretend to explain the origin of biological homochirality. All tested models are available in the folder models that come with Listanalchem. Some models are stable and incapable of producing SMSB; others are unstable and exhibit SMSB. All those models with their variations can be studied efficiently and in a short time by changing, adding, or removing reactions to make the models fit thermodynamic and kinetics principles; or by trying other changes that an interested user can imagine. This makes it easy to verify or refute the claims about different models that have been or are being published in the specialized literature.

Listanalchem is free/open software that anyone can download and use. The code and the installation instructions are on https://gitlab.com/homochirality/listanalchem.

## Declaration of interests

The authors declare that they have no known competing financial interests or personal relationships that could have appeared to influence the work reported in this paper.

## CRediT author statement

**Elkin Cruz:** Conceptualization, Methodology, Software implementation, Writing - original draft. **Andrés Montoya**: Algorithms development, Conceptualization, Writing-Reviewing, Editing. **Jesús Ágreda:** Conceptualization, Methodology, some details in the Software implementation, Validity tests, Writing-Reviewing.

## Supplementary material

The method presented here is available on https://gitlab.com/homochirality/listanalchem as the python program Listanalchem.

## Data Availability

No data was used for the research described in the article.

## Footnotes

1. Observe that the French word mineurs is used instead of the English word "minors" to avoid reserved word problems in the python implementation. In the rest of the text, both mineurs and minors will be used as synonymous.

2 Velocity Function is here in the transposed form to save space.
